# Coronary Flow Velocity Reserve Using Dobutamine Test for Noninvasive Functional Assessment of Myocardial Bridging

**DOI:** 10.3390/jcm11010204

**Published:** 2021-12-30

**Authors:** Srdjan B. Aleksandric, Ana D. Djordjevic-Dikic, Vojislav L. Giga, Milorad B. Tesic, Ivan A. Soldatovic, Marko D. Banovic, Milan R. Dobric, Vladan Vukcevic, Miloje V. Tomasevic, Dejan N. Orlic, Nikola Boskovic, Ivana Jovanovic, Milan A. Nedeljkovic, Goran Stankovic, Miodrag C. Ostojic, Branko D. Beleslin

**Affiliations:** 1 Cardiology Clinic, University Clinical Center of Serbia, 11000 Belgrade, Serbia; skali.ana7@gmail.com (A.D.D.-D.); voja2011@yahoo.com (V.L.G.); misa.tesic@gmail.com (M.B.T.); markobanovic71@gmail.com (M.D.B.); iatros007@gmail.com (M.R.D.); vladan.vukcevic@gmail.com (V.V.); milojetomasevic@gmail.com (M.V.T.); orlicmail@yahoo.com (D.N.O.); belkan87@gmail.com (N.B.); ivana170679@gmail.com (I.J.); milanned@hotmail.com (M.A.N.); gorastan@gmail.com (G.S.); branko.beleslin@gmail.com (B.D.B.); 2 Faculty of Medicine, University of Belgrade, 11000 Belgrade, Serbia; soldatovic.ivan@gmail.com (I.A.S.); mostojic2011@gmail.com (M.C.O.); 3 Institute of Medical Statistics and Informatics, Faculty of Medicine, University of Belgrade, 11000 Belgrade, Serbia; 4 Department of Internal Medicine, Faculty of Medical Sciences, University of Kragujevac, 34000 Kragujevac, Serbia; 5 Institute for Cardiovascular Diseases Dedinje, 11000 Belgrade, Serbia

**Keywords:** myocardial bridging, myocardial ischemia, stress-echocardiography, coronary flow velocity reserve, transthoracic Doppler echocardiography, dobutamine

## Abstract

Background: It has been shown that coronary flow velocity reserve (CFVR) measurement by transthoracic Doppler echocardiography (TTDE) during dobutamine (DOB) provocation provides a more accurate functional evaluation of myocardial bridging (MB) compared to adenosine. However; the cut-off value of CFVR during DOB for identification of MB associated with myocardial ischemia has not been fully clarified. Purpose: This prospective study aimed to determine the cut-off value of TTDE-CFVR during DOB in patients with isolated-MB, as compared with stress-induced wall motion abnormalities (VMA) during exercise stress-echocardiography (SE) as reference. Methods: Eighty-one symptomatic patients (55 males [68%], mean age 56 ± 10 years; range: 27–74 years) with the existence of isolated-MB on the left anterior descending artery (LAD) and systolic MB-compression ≥50% diameter stenosis (DS) were eligible to participate in the study. Each patient underwent treadmill exercise-SE, invasive coronary angiography, and TTDE-CFVR measurements in the distal segment of LAD during DOB infusion (DOB: 10–40 μg/kg/min). Using quantitative coronary angiography, both minimal luminal diameter (MLD) and percent DS at MB-site at end-systole and end-diastole were determined. Results: Stress-induced myocardial ischemia with the occurrence of WMA was found in 23 patients (28%). CFVR during peak DOB was significantly lower in the SE-positive group compared with the SE-negative group (1.94 ± 0.16 vs. 2.78 ± 0.53; *p* < 0.001). ROC analyses identified the optimal CFVR cut-off value ≤ 2.1 obtained during high-dose dobutamine (>20 µg/kg/min) for the identification of MB associated with stress-induced WMA, with a sensitivity, specificity, positive and negative predictive value of 96%, 95%, 88%, and 98%, respectively (AUC 0.986; 95% CI: 0.967–1.000; *p* < 0.001). Multivariate logistic regression analysis revealed that MLD and percent DS, both at end-diastole, were the only independent predictors of ischemic CFVR values ≤2.1 (OR: 0.023; 95% CI: 0.001–0.534; *p* = 0.019; OR: 1.147; 95% CI: 1.042–1.263; *p* = 0.005; respectively). Conclusions: Noninvasive CFVR during dobutamine provocation appears to be an additional and important noninvasive tool to determine the functional severity of isolated-MB. A transthoracic CFVR cut-off ≤2.1 measured at a high-dobutamine dose may be adequate for detecting myocardial ischemia in patients with isolated-MB.

## 1. Introduction

Coronary flow velocity reserve (CFVR) measured by transthoracic Doppler echocardiography (TTDE) is a clinically useful and feasible noninvasive diagnostic index for the assessment of fixed coronary stenosis severity [1,2,3,4,5,6,7,8]. Although adenosine-induced maximal hyperemia is considered a gold standard for TTDE-CFVR measurements in the functional assessment of fixed coronary stenosis, the functional assessment of myocardial bridging (MB) is less reliable [9,10,11,12,13,14]. Despite being considered a benign coronary lesion, MB may cause life-threatening events such as ventricular arrhythmias, acute coronary syndrome, or even sudden cardiac death [15,16,17,18]. As a result of these events, concerns have been raised regarding its clinical significance and the need for appropriate diagnostic test/s which could be helpful in the identification of MB associated with myocardial ischemia [14]. Therefore, previous studies indicate that the functional assessment of MB should include dobutamine provocation with possibly exaggerated extravascular systolic coronary artery compression [9,10,11,12,13,14]. Additionally, the feasibility of assessing CFVR by TTDE during dobutamine provocation is high and the steady-state hyperemia induced with a high-doses of dobutamine (>20 µg/kg/min) is similar to adenosine-induced one [13,14,19,20,21,22,23,24,25,26]. Recently, it has been demonstrated that TTDE-CFVR measured at high-dose dobutamine provides a more accurate functional evaluation of MB compared to adenosine [13]. Still, CFVR obtained by TTDE has not been thoroughly evaluated to determine whether it is useful for functional assessment of MB severity. Therefore, the purpose of the study was to evaluate the adequate cut-off value of TTDE-CFVR during dobutamine provocation, as compared with exercise-induced myocardial ischemia as reference.

## 2. Methods

### 2.1. Study Population

Eighty-one symptomatic patients (55 males [68%], mean age 56 ± 10 years, range: 27–74 years) undergoing coronary angiography due to recurrent typical or atypical chest pain were eligible to participate in this prospective study. Key inclusion criteria, in addition to chest pain, was the existence of isolated-MB on the left anterior descending artery (LAD) and systolic MB-compression ≥50% diameter stenosis (DS), after intracoronary administration of 200 µg of nitroglycerin (NTG), as measured by quantitative coronary angiography (QCA). Exclusion criteria were: (1) patients aged ≤18 years old; (2) presence of coronary artery disease in the LAD and/or other coronary arteries; (3) acute coronary syndrome with or without ST elevation; (4) any previous myocardial infarction; (5) any previous percutaneous or surgical myocardial revascularization; (6) left ventricular ejection fraction (LVEF) <40%; (7) left ventricular (LV) hypertrophy; (8) hypertrophic, dilated, or restrictive cardiomyopathies; (9) any valvular heart disease; (10) uncontrolled arterial hypertension; (11) atrial fibrillation (paroxysmal, persistent, or permanent); and (12) renal failure (acute or chronic).

### 2.2. Study Protocol

Each patient underwent standard two-dimensional echocardiography, treadmill-exercise stress echocardiography (SE), computed-tomography coronary angiography (CTCA), invasive coronary angiography, and TTDE-CFVR measurements during dobutamine provocation which was performed within 1 month after the invasive procedure. Study patients without objective signs of myocardial ischemia on exercise-SE were referred to CTCA in order to rule-out the presence of coronary lesion/s (fixed coronary stenosis, coronary ectasia, or MB) as a possible cause of chest pain. If CTCA showed the presence of intramyocardial LAD segment penetrating the interventricular septum at a depth of at least 1.0 mm from the anterior interventricular groove surface (deep type of MB) and no fixed stenosis on any epicardial coronary artery, then invasive coronary angiography was performed. Antianginal drugs (beta-blockers, calcium-channel blockers, long-acting nitrates, trimetazidine, and ranolazine) and xanthine-containing foods or beverages (coffee, tea, sugar drinks, sweets, chocolate, and fruits), were discontinued 24 to 48 h before exercise-SE, invasive coronary angiography and TTDE-CFVR measurements, separately. The study protocol was approved by the Ethical Committees of University Clinical Center of Serbia and Faculty of Medicine University of Belgrade (Belgrade, Serbia). Informed consent was obtained from all patients.

### 2.3. Exercise Stress-Echocardiography

Following a standard two-dimensional echocardiography examination, all patients underwent treadmill exercise-SE according to the maximal Bruce protocol, as previously described [13,27,28]. Briefly, a senior echocardiographer blinded to patients’ clinical, angiographic, and functional status analyzed and interpreted echocardiographic images using a cardiovascular ultrasound system (Acuson Sequoia C256, Siemens Medical Solutions USA, Mountain View, CA, USA) with a 3V2C multifrequency transducer using second-harmonic technology. Based on the 17-segment model, the segmental walls motion in the LV was categorized as follows: 1 = normal, 2 = hypokinetic, 3 = akinetic, and 4 = dyskinetic [29]. Myocardial ischemia was deemed to exist when new wall-motion abnormalities (WMA) appeared in at least two adjacent LV segments in the LAD territory during the exercise-SE test.

### 2.4. Quantitative Coronary Angiography

Invasive coronary angiography and detailed frame-by-frame QCA analysis of the interpolated reference diameter, minimal luminal diameter (MLD), and percent DS, at the most severe MB-site, were performed as previously described in detail elsewhere [14,30]. Briefly, all angiographic images were obtained at least 1 min after ic. administration of 200 µg of NTG and analyzed in the same views and with the same positioning of the cursor at both end-systole (peak of T wave) and end-diastole (beginning of QRS complex). A senior investigator blinded to the patients’ clinical, functional, and echocardiographic status analyzed the images offline using a QCA system (Siemens Quantcor QCA, Siemens Healthineers AG, Erlangen, Germany).

### 2.5. Coronary Flow Velocity Reserve Measurements by Transthoracic Doppler Echocardiography

The same ultrasound unit was used to assess CFVR during dobutamine provocation, as had been described in detail previously [13]. To adequately measure CFVR at peak dobutamine dose, the goal was to achieve an increase in heart rate (HR) ≥50 bpm from baseline and/or a HR ≥75% of the maximum predicted for age [31]. Before and during testing, systemic hemodynamic parameters (HR and blood pressure) and 12-lead ECG were continuously monitored. The rate-pressure product (RPP) was computed as HR × systolic-BP (bpm × mmHg). At the end of the procedure, metoprolol iv. (2.5–5.0 mg over 1–2 min) were administrated until the normalization of HR (50–80 bpm).

**Figure 1:**
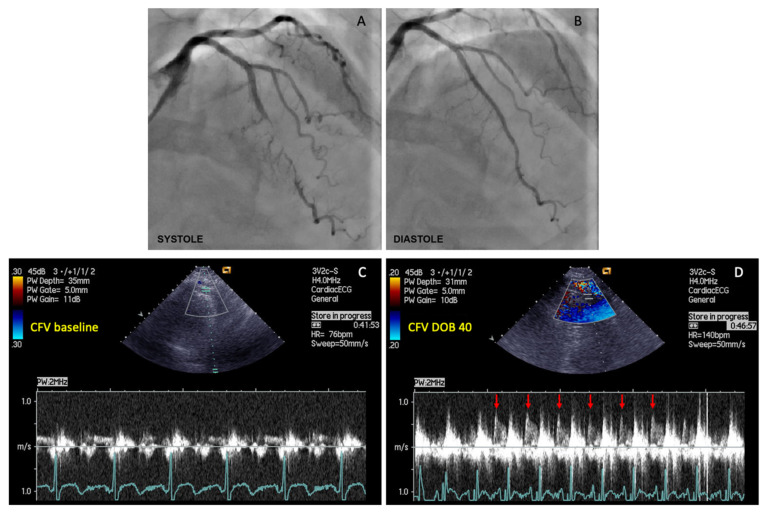
The example of coronary flow velocity reserve (CFVR) measurements obtained by transthoracic Doppler echocardiography (TTDE) in the left anterior descending (LAD) artery distal to the myocardial bridging (MB), before and during iv. infusion of high-dose dobutamine (40 µg/kg/min). (**A**) Coronary angiography revealed myocardial bridging (MB) with severe intramyocardial LAD segment compression (>90% diameter stenosis) during systole; (**B**) Coronary angiography showed a significant decompression of intramyocardial LAD segment during diastole in the same patient; (**C**) CFV measurement under basal conditions (CFV baseline), and (**D**) CFV measurement at peak dobutamine dose (CFV DOB 40). The heart rate under basal conditions was 76 bpm, while during peak dobutamine infusion was 140 bpm (delta-HR 64 bpm). Coronary flow velocity reserve equals 2.96 (CFV DOB 40/CFV baseline = 0.77/0.26 = 2.96). Red arrows showing characteristic diastolic CFV profile during peak dobutamine infusion in the LAD distal to the MB. This phenomenon is characterized by an abrupt acceleration followed by rapid deceleration of the CFV at early-diastole and flow plateau during mid-to-late diastole (“finger-tip” phenomenon). CFV = coronary flow velocity; DOB 40 = dobutamine at 40 µg/kg/min.

Maximal (peak) diastolic coronary flow velocities (CFVs) under basal conditions and during maximal hyperemia induced by peak dobutamine infusion (30 or 40 µg/kg/min) were measured in the distal segment of LAD, by a senior investigator who was blinded for the patient’s clinical and functional status (Figure 1). CFVR was calculated as the ratio between peak diastolic CFV obtained during peak dobutamine infusion and peak diastolic CFV under basal conditions.

### 2.6. Statistical Analysis

Statistical analysis of the data was performed using IBM SPSS Statistics for Windows, version 26.0 (IBM Corporation, Armonk, NY, USA). Categorical variables are presented as counts with percentages and continuous variables as means ± standard deviations (SD). Kolmogorov–Smirnov and Levene tests were used to verify the normal distribution of continuous variables. Differences between categorical variables were analyzed using a Chi-squared or Fischer’s exact test, depending on group size, whereas those between continuous variables were analyzed using a Student’s *t*-test (paired or unpaired) as appropriate. A Pearson correlation coefficient, along with a linear regression model, is used to estimate the correlation between continuous variables. ANOVA for repeated measures was used to determine differences in hemodynamic parameters measured under basal conditions and during peak dobutamine infusion. To assess the most appropriate cut-off value of CFVR during peak dobutamine infusion for stratifying MB-patients with and without stress-induced myocardial ischemia, receiver operating characteristic (ROC) curve with 95% confidence intervals (CI) and the highest Youden’s index (sensitivity + specificity-1) was used. Cohen’s kappa coefficient was applied to evaluate the agreement between two dichotomous variables: exercise stress-induced myocardial ischemia (0 = absent; 1 = present), and TTDE-CFVR (0 = CFVR greater than cut-off value; 1 = CFVR less than/equal to cut-off value). Univariate binary logistic regression was used to identify whether different variables are significantly associated with established ischemic CFVR values. To establish variables independently associated with ischemic CFVR values in MB-patients, univariate predictors with a value of *p* < 0.2 were included in a multivariate regression model using a backward method. Statistical significance was defined as a two-sided value of *p* < 0.05.

## 3. Results

Mean systolic compression of the intramyocardial LAD segment was 62 ± 10% (range: 51–95% DS). At peak dobutamine dose, the HR ≥50 bpm from baseline was achieved in all patients (100%), whereas the HR ≥75% of maximum age-predicted HR was achieved in 73 patients (90%). However, due to the onset of chest pain and/or ischemic ECG changes, the remaining 8 patients (10%) were not able to reach HR ≥75% of maximum age-predicted HR during dobutamine provocation, requiring an earlier termination of the test. There were no major complications. Dobutamine provocation caused ventricular bigeminy in one patient, which was resolved after its cessation and concomitantly administering metoprolol (2.5–5.0 mg).

Stress-induced myocardial ischemia with the occurrence of WMA in medial and distal segments of the anterior septum was found in 23 patients (28%). Thirteen of 23 patients with WMA and 1 of 58 patients without WMA had ST depressions ≥1.0 mm during exercise (56.5 vs. 1.7%, *p* < 0.001). Exercise-induced chest pain was experienced by 11 of 23 patients with VMA and 11 of 58 patients without VMA (47.8 vs. 19.0%, *p* = 0.013).

Table 1 and Table 2 summarize demographic, clinical, and angiographic characteristics of MB-patients regarding the presence of stress-induced VMA during exercise-SE. In terms of demographic and clinical variables, no significant differences were found between SE-positive and SE-negative groups. At end-diastole, percent DS was significantly higher, and MLD significantly lower in SE-positive in comparison to SE-negative group (39 ± 8 vs. 27 ± 8%, *p* < 0.001; 1.55 ± 0.31 vs. 1.96 ± 0.36 mm, *p* < 0.001, respectively), but not at end-systole (64 ± 12 vs. 62 ± 10%, *p* = 0.496; 0.89 ± 0.34 vs. 1.01 ± 0.29 mm, *p* = 0.200, respectively).

### 3.1. Coronary Physiological Parameters during Dobutamine Provocation: Relation to Stress-Induced VMA

**Figure 2:**
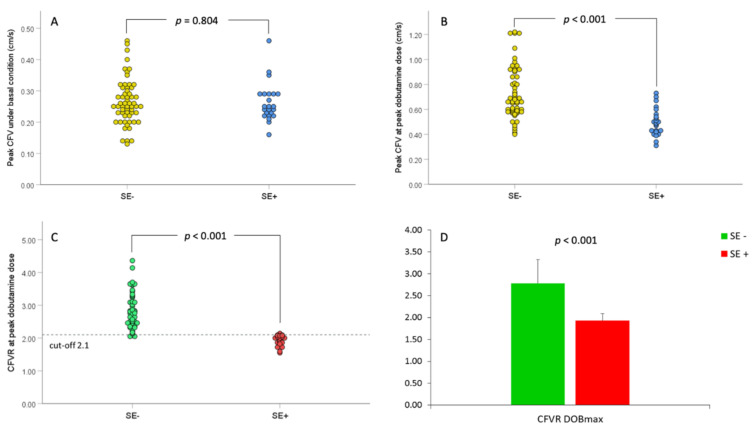
Peak coronary flow velocity (CFV) and coronary flow velocity reserve (CFVR) before and during peak dobutamine dose (DOBmax) in relation to stress-echocardiography (SE) results. (**A**). Scatterplot of peak CFV values under baseline conditions in relations to SE-results; (**B**). Scatterplot of peak CFV values during DOBmax in relations to SE-results; (**C**). Scatterplot of CFVR values in relation to SE-results, and (**D**). Bar graphs of mean CFVR values before and DOBmax in relation to SE-results. The dashed line in panel C represents the ischemic thresholds for CFVR at peak dobutamine dose (2.1). SE− = group of patients without stress-induced ischemia; SE+ = group of patients with stress-induced ischemia.

Table 3 shows systemic and coronary physiological parameters before and after peak dobutamine infusion regarding the presence of stress-induced VMA during exercise-SE in MB-patients. There were no differences in HR, both systolic and diastolic BP, and RPP before and after peak dobutamine infusion between SE-positive and SE-negative groups. However, both CFV and CFVR after peak dobutamine infusion were significantly lower in SE-positive in comparison to SE-negative group (50.78 ± 11.05 vs. 72.62 ± 20.74 cm/s, *p* < 0.001; 1.93 ± 0.16 vs. 2.78 ± 0.54%, *p* < 0.001, respectively) (Table 3, Figure 2).

### 3.2. Diagnostic Value of Coronary Flow Velocity Reserve with Stress-Induced VMA as the Reference Standard

**Figure 3:**
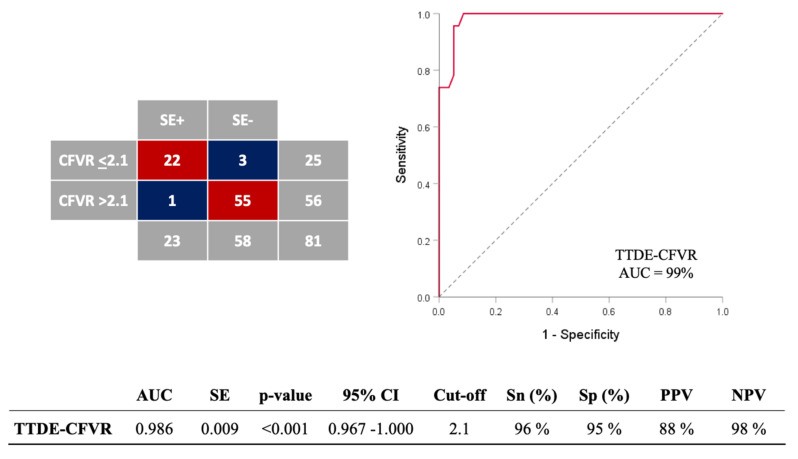
ROC analysis for assessing the accuracy of coronary flow velocity reserve (CFVR) obtained by transthoracic Doppler echocardiography (TTDE) for detection of stress-induced wall-motion abnormalities (VMA) in MB-patients. The overall diagnostic value of the test was 95% (77/81). ROC = receiver-operating characteristics curve; AUC = area under curve; SE = standard error; CI = confidence interval; Sn = sensitivity; Sp = specificity; PPV = positive predictive value; NPV = negative predictive value. DOBmax = peak dobutamine infusion.

ROC analyses identified the optimal CFVR cut-off value ≤ 2.1 obtained during high-dose dobutamine (>20 µg/kg/min) for the identification of MB associated with stress-induced ischemia, with a sensitivity, specificity, positive and negative predictive value of 96%, 95%, 88%, and 98%, respectively (AUC 0.986, 95% CI: 0.967–1.000, *p* < 0.001) (Figure 3). The overall diagnostic value of the test was 95%. The classification agreement between dichotomized values of two categorical variables (TTDE-CFVR: 0 = >2.1; 1 = ≤ 2.1; SE-results: 0 = without stress-induced ischemia, 1 = with stress-induced ischemia) was high (kappa value = 0.882, *p* < 0.001).

### 3.3. Coronary Physiological Parameters during Dobutamine Provocation: Relation to Angiographic Data

**Figure 4:**
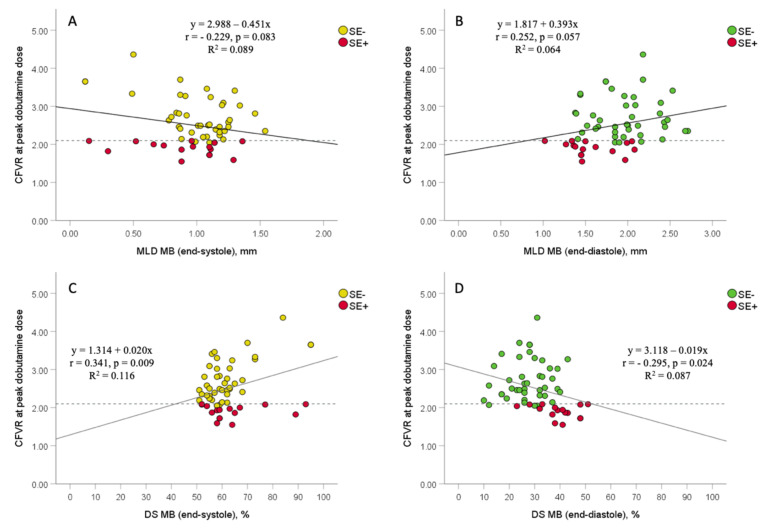
The association between coronary flow velocity reserve (CFVR) obtained by transthoracic Doppler echocardiography (TTDE) and angiographic data (minimal luminal diameter and percent diameter stenosis) in MB-patients, with regards to stress-echocardiography (SE) results. Scatterplots between CFVR and both MLD at end-systole and end-diastole (panels (**A**,**B**)), and between CFVR and both percent DS at end-systole and end-diastole (panels (**C**,**D**)). Dashed lines represent the ischemic thresholds for CFVR at peak dobutamine dose (2.1), while solid lines represent a linear regression line. MB = myocardial bridging; MLD = minimal luminal diameter; DS = diameter stenosis; SE− = group of patients without stress-induced ischemia; SE+ = group of patients with stress-induced ischemia.

There were a significant correlations between CFVR and both percent DS at end-systole and end-diastole after peak dobutamine infusion (r = 0.341, *p* = 0.009; r= −0.295, *p* = 0.024, respectively), and a borderline correlation between CFVR and MLD at end-systole and end-diastole (r= −0.229, *p* = 0.083; r = 0.252, *p* = 0.057, respectively) (Figure 4). However, multivariate logistic regression analyses showed that MLD and percent DS at MB-site, both at end-diastole, were the only independent predictors of ischemic CFVR values (≤2.1) measured after peak dobutamine dose in MB-patients (OR: 0.023, 95%: 0.001–0.534, *p* = 0.019; OR: 1.147, 95% CI: 1.042–1.263; *p* = 0.005, respectively) (Table 4). MLD and percent DS at end-diastole were not analyzed together in the same multivariate analysis due to their high correlation and multicollinearity (r= −0.667, *p* < 0.001).

## 4. Discussion

The present study evaluated for the first time the best cut-off value of CFVR during dobutamine provocation to identify the presence of myocardial ischemia in patients with MB, as compared to the results of exercise-SE test. The major finding of this study is that CFVR during inotropic stimulation with high-doses of dobutamine is feasible, safe, reliable, and clinically useful noninvasive test for physiologic assessment of the MB severity. The optimal cut-off of ≤2.1 for CFVR at peak dobutamine dose, as determined by TTDE, has the best sensitivity, specificity, positive and negative predictive value, for identifying the MB concerning exercise stress-induced myocardial ischemia. The same cut-off of ≤2.1 for TTDE-CFVR during high-dose dobutamine provocation was suggested in the earlier study by Takeuchi et al. regarding functional evaluation of fixed coronary stenosis [21]. This was the only study, so far, that evaluated CFVR values at peak dobutamine dose obtained by TTDE in the context of dobutamine-induced WMA in the LAD territory. This study showed that CFVR during dobutamine provocation with the ischemic threshold of ≤2.1 had a sensitivity, specificity, positive and negative predictive value of 92%, 86%, 73%, and 96%, respectively, for detecting the presence of dobutamine-induced myocardial ischemia. Additionally, three other studies which previously compared the value of CFVR during adenosine provocation obtained by TTDE or invasive intracoronary Doppler guidewire, with the results of either dobutamine-SE or single-photon emission computed tomography (SPECT) in patients with fixed LAD stenosis of intermediate severity, demonstrated that CFVR ≤2.0 had similar sensitivity (88–94%), specificity (89–95%), positive and negative predictive values (64–94% and 95–97%, respectively), for detecting ischemia on noninvasive tests [6,21,32]. Accordingly, our study reinforces previous findings that myocardial ischemia is a unique phenomenon, characterized by different pathophysiological mechanisms in different types of coronary lesions (MB versus fixed stenosis) [14,33]. Similar findings were also presented in studies that compared invasive physiological indices (conventional and diastolic fractional flow reserve, as well as instantaneous wave-free ratio) obtained during both adenosine and dobutamine provocation against the results of noninvasive provocation tests for myocardial ischemia in MB-patients [10,12,14]. Therefore, from both clinical and pathophysiological views, dobutamine seems to be the agent of choice for functional evaluation of the MB severity.

The present study showed a high negative predictive value for the CFVR threshold of 2.1 (98%) which is in agreement with previous studies that evaluated the value of CFVR measured either with adenosine or dobutamine provocation for the prediction of myocardial ischemia on noninvasive tests [6,21,22,32]. In clinical settings, it means that CFVR > 2.1 at peak dobutamine dose could safely exclude the presence of functionally significant MB on LAD.

Our study demonstrates that both percent DS and MLD at end-diastole, but not at end-systole, were independent predictors of ischemic CFVR values ≤ 2.1 during dobutamine provocation, suggesting that coronary blood flow in a coronary artery with MB mainly depends on the severity of residual MB-compression during diastole. Similarly, our previous study in MB-patients evaluating the role of invasive conventional and diastolic fractional flow reserve measurement during both adenosine and dobutamine provocation has also observed that percent DS at end-diastole and diastolic fractional flow reserve at peak dobutamine dose were the only independent predictors of stress-induced myocardial ischemia [14]. The present study with noninvasive CFVR measured at peak dobutamine dose also supported previous findings by Escaned et al. that MB primarily altered diastolic hemodynamics by generating a significant diastolic pressure gradient across the MB during dobutamine provocation [10,14]. Furthermore, according to studies using QCA and intravascular ultrasound, delayed early diastolic relaxation of the intramyocardial arterial segment is the main hemodynamic disorder of MB resulting in incomplete MB-decompression at mid-to-late diastole [30,34,35,36,37,38]. Based on these findings, it is reasonable to assume that stress-induced myocardial ischemia in MB-patients is caused by prolonged early diastolic artery relaxation accompanied by persistent reductions in vessel luminal diameter in mid-to-late diastole, which may deteriorate during dobutamine or exercise stress testing due to tachycardia and the consequent shortening of the diastolic perfusion time [30,34,35,36,37,38].

### Study Limitations

Even though exercise-SE is a well-established diagnostic tool in detecting myocardial ischemia, due to its semi-quantitative nature and small areas of WMA, certain patients might be misdiagnosed regarding the presence of myocardial ischemia [28]. However, due to the close relationship between the degree of myocardial ischemia and clinical outcome, and the high sensitivity of exercise-SE, the presence of mild ischemia carries a favorable prognosis [39]. In addition, specifically in these patients suggestive of myocardial ischemia, noninvasive evaluation of CFVR during dobutamine provocation may further classify and risk-stratify MB-patients.

As the most simple and reproducible variable, CFVR, defined as the ratio of hyperemic to basal CFV during diastole, was used in our study since it correlates strongly with coronary perfusion reserve determined by PET [40]. Since the diastolic flow velocities obtained by TTDE depend on the positioning of ultrasound beam, we performed angle correction when necessary.

## 5. Conclusions

Noninvasive CFVR during dobutamine provocation appears to be an additional and important noninvasive tool to determine the functional severity of isolated-MB. A transthoracic CFVR cut-off ≤2.1 measured at a high-dobutamine dose may be adequate for detecting myocardial ischemia in patients with isolated-MB.

## Figures and Tables

**Table 1:** Demographic and clinical characteristics of MB-patients regarding the presence of stress-induced wall-motion abnormalities (VMA) in the LAD territory.

Variable	All (*n* = 81)	SE − (*n* = 58)	SE + (*n* = 23)	*p*-Value
Age ± SD, years	56 ± 10	55 ± 10	57 ± 9	0.275
Gender, males (%)	55 (68)	38 (65)	17 (74)	0.466
BMI ± SD, kg/m^2^	27.0 ± 3.9	27.1 ± 4.1	26.5 ± 3.5	0.526
Hypertension, *n* (%)	59 (73)	45 (78)	14 (61)	0.127
Diabetes, *n* (%)	10 (12)	7 (12)	3 (13)	0.904
Smoking, *n* (%)	37 (46)	26 (45)	11 (48)	0.807
Hyperlipidemia, *n* (%)	59 (73)	44 (76)	15 (65)	0.331
Family history, *n* (%)	49 (60)	33 (57)	16 (70)	0.293
LVEF ± SD, %	64 ± 8	64 ± 8	62 ± 7	0.161
Typical chest pain, *n* (%)	44 (54)	29 (50)	15 (65)	0.215
Atypical chest pain, *n* (%)	37 (46)	29 (50)	8 (35)	0.215

Data are expressed as mean ± SD or as number (%). SE− = group of MB-patients without stress-induced VMA; SE+ = group of MB-patients with stress-induced VMA. BMI = body-mass index; LAD = left anterior descending artery; LVEF = left ventricle ejection fraction; MB = myocardial bridging.

**Table 2:** Angiographic characteristics of MB-patients regarding the presence of stress-induced wall-motion abnormalities (VMA) in the LAD territory.

Variable	All (*n* = 81)	SE− (*n* = 58)	SE + (*n* = 23)	*p*-Value
RD (end-systole) ± SD, mm	2.59 ± 0.39	2.64 ± 0.40	2.47 ± 0.33	0.126
RD (end-diastole) ± SD, mm	2.65 ± 0.40 *	2.70 ± 0.40 *	2.53 ± 0.36 *	0.140
MLD (end-systole) ± SD, mm	0.98 ± 0.31	1.01 ± 0.29	0.89 ± 0.34	0.200
MLD (end-diastole) ± SD, mm	1.84 ± 0.39 *	1.96 ± 0.36 *	1.55 ± 0.31 *	<0.001
DS (end-systole) ± SD, %	62 ± 11	62 ± 10	64 ± 12	0.496
DS (end-diastole) ± SD, %	31 ± 10 *	27 ± 8 *	39 ± 8 *	<0.001

Data are expressed as mean ± SD or as number (%). SE− = group of MB-patients without stress-induced VMA; SE+ = group of MB-patients with stress-induced VMA. DS = diameter stenosis; LAD = left anterior descending artery; MB = myocardial bridging; MLD = minimal luminal diameter; RD = reference diameter. * *p* < 0.05 vs. end-systole.

**Table 3:** Systemic and coronary physiological parameters before and after peak dobutamine infusion regarding the presence of stress-induced wall-motion abnormalities (VMA) in MB-patients.

	All (*n* = 81)	SE− (*n* = 58)	SE + (*n* = 23)	*p*-Value
HR, bpm (baseline)	74 ± 11	75 ± 10	72 ± 11	0.240
HR, bpm (DOBmax)	139 ± 9 *	140 ± 8 *	136 ± 12 *	0.093
Mean systolic BP, mmHg (baseline)	129 ± 13	130 ± 12	126 ± 13	0.253
Mean systolic BP, mmHg (DOBmax)	133 ± 17	134 ± 18	129 ± 14	0.264
Mean diastolic BP, mmHg (baseline)	82 ± 10	82 ± 10	82 ± 10	0.946
Mean diastolic BP, mmHg (DOBmax)	82 ± 9	83 ± 9	80 ± 7	0.141
RPP, ×10^3^ bpm∙mmHg (baseline)	9.6 ± 2.0	9.7 ± 1.8	9.1 ± 2.2	0.186
RPP, ×10^3^ bpm∙mmHg (DOBmax)	18.5 ± 2.6 *	18.8 ± 2.4 *	17.6 ± 2.9 *	0.151
CFV, cm/s (baseline)	26.42 ± 6.97	26.47 ± 7.28	26.30 ± 6.28	0.804
CFV, cm/s (DOBmax)	67.44 ± 21.15 *	72.62 ± 20.74 *	50.78 ± 11.05 *	<0.001
CFVR	2.55 ± 0.61	2.78 ± 0.54	1.93 ± 0.16	<0.001

Data are expressed as mean ± SD. SE− = group of MB-patients without stress-induced VMA; SE+ = group of MB-patients with stress-induced VMA. BL = basal conditions, before dobutamine infusion; BP = blood pressure; CFV = coronary flow velocity; CFVR = coronary flow velocity reserve; DOBmax = peak dobutamine dose; HR = heart rate; RPP = rate-pressure product; * *p* < 0.05 vs. baseline.

**Table 4:** Multivariate logistic regression analyses for all significant univariate variables (*p* ≤ 0.2) predicting ischemic CFVR value ≤ 2.1 in MB-patients.

Univariate Analysis	OR (95% CI for OR)	*p*-Value	R^2^	HL Test *p*-Value
Age, years	1.037 (0.982–1.094)	0.182	0.033	0.411
Gender, males	1.759 (0.604–5.120)	0.300	0.019	1.000
BMI, kg/m^2^	0.954 (0.841–1.082)	0.461	0.010	0.975
Hypertension	0.537 (0.193–1.499)	0.235	0.024	1.000
Diabetes	1.587 (0.406–6.209)	0.507	0.007	1.000
Smoking	1.444 (0.561–3.722)	0.446	0.010	1.000
Heredity	1.239 (0.467–3.284)	0.667	0.003	1.000
Hyperlipidemia	0.537 (0.193–1.499)	0.235	0.024	1.000
LVEF, %	0.950 (0.888–1.016)	0.134	0.045	0.532
Typical chest pain, %	1.778 (0.674–4.691)	0.245	0.024	1.000
Atypical chest pain, %	0.563 (0.213–1.484)	0.245	0.024	1.000
RD (end-systole), mm	0.326 (0.073–1.454)	0.142	0.054	0.666
RD (end-diastole), mm	0.340 (0.078–1.481)	0.151	0.051	0.223
MLD MB (end-systole), mm	0.426 (0.074–2.465)	0.341	0.022	0.080
MLD MB (end-diastole), mm	0.072 (0.012–0.459)	0.005	0.218	0.931
DS MB (end-systole), %	1.009 (0.958–1.062)	0.745	0.003	0.189
DS MB (end-diastole), %	1.145 (1.053–1.244)	0.001	0.308	0.589
**Model 1. Backward Method with Age, LVEF, RD, and MLD MB at End-Diastole:**	**OR (95% CI for OR)**	***p*-Value**	**R^2^**	**HL Test *p*-Value**
Age, years	1.043 (0.970–1.122)	0.252	0.314	0.224
LVEF, %	0.936 (0.856–1.024)	0.152	0.314	0.224
RD (end-diastole), mm	5.321 (0.340–83.172)	0.233	0.314	0.224
MLD MB (end-diastole), mm ^a^	0.023 (0.001–0.534)	0.019	0.314	0.224
**Model 2. Backward Method with Age, LVEF, RD, and %DS MB at End-diastole:**	**OR (95% CI for OR)**	***p*-Value**	**R^2^**	**HL Test *p*-Value**
Age, years	1.059 (0.978–1.146)	0.161	0.395	0.533
LVEF, %	0.939 (0.853–1.033)	0.197	0.395	0.533
RD (end-diastole), mm	0.438 (0.076–2.519)	0.355	0.395	0.533
DS MB (end-diastole), % ^a^	1.147 (1.042–1.263)	0.005	0.395	0.533

Dependent variable: ischemic CFVR value ≤ 2.1 in the LAD territory. Multivariate logistic regression analyses were adjusted for all variables with *p* ≤ 0.2 in univariate analysis. ^a^ only variable in the model. CI = confidence interval; R^2^ = Nagelkerke R square, HL = Hosmer and Lemeshow test. MB = myocardial bridging; MLD = minimal luminal diameter; DS = diameter stenosis; RD = reference diameter.

## Data Availability

The data presented in this study are available on request from the corresponding author. The data are not publicly available due to privacy or ethical restrictions.

## Abbreviations

CFV	coronary flow velocity
CFVR	coronary flow velocity reserve
DOB	dobutamine
DS	diameter stenosis
MB	myocardial bridging
MLD	minimal luminal diameter
LAD	left anterior descending coronary artery
SE	stress-echocardiography
QCA	quantitative coronary angiography
RD	reference diameter
WMA	wall-motion abnormalities
